# Naturally activated adaptive immunity in COVID‐19 patients

**DOI:** 10.1111/jcmm.15771

**Published:** 2020-09-25

**Authors:** Xiaofeng Yang, Tongxin Dai, Xiaobo Zhou, Hongbo Qian, Rui Guo, Lei Lei, Xingzhe Zhang, Dan Zhang, Lin Shi, Yanbin Cheng, Jinsong Hu, Yaling Guo, Baojun Zhang

**Affiliations:** ^1^ Department of Pathogenic Microbiology and Immunology School of Basic Medical Sciences Xi'an Jiaotong University Health Science Center Xi'an, Shaanxi China; ^2^ Department of Clinical Laboratory The 8th hospital of Xi'an Xi'an, Shaanxi China; ^3^ Key Laboratory of Shaanxi Province for Craniofacial Precision Medicine Research College of Stomatology Xi’an Jiaotong University Xi'an, Shaanxi China; ^4^ Department of Cell Biology and Genetics School of Basic Medical Sciences Xi'an Jiaotong University Health Science Center Xi'an, Shaanxi China; ^5^ Key Laboratory of Environment and Genes Related to Diseases (Xi'an Jiaotong University) Ministry of Education Xi'an, Shaanxi China; ^6^ Institute of infection and immunity Translational Medicine Institute Xi’an Jiaotong University Health Science Center Xi’an, Shaanxi China; ^7^ Basic and Translational Research Laboratory of Immune Related Diseases Xi’an, Shaanxi China

**Keywords:** adaptive immunity, COVID‐19, lymphocyte, SARS‐CoV‐2

## Abstract

Coronavirus disease‐2019 (COVID‐19) caused by severe acute respiratory syndrome coronavirus (SARS‐CoV‐2) has rapidly spread worldwide, threatening the health and lives of many people. Unfortunately, information regarding the immunological characteristics of COVID‐19 patients remains limited. Herein, we collected blood samples from 18 healthy donors (HDs) and 38 COVID‐19 patients to analyse changes in the adaptive immune cell populations and their phenotypes. We observed that the lymphocyte percentage moderately decreased, CD4 and CD8 T cell percentage among lymphocytes were similar, and B cell percentage was increased in COVID‐19 patients in comparison to that in HDs. T cells, especially CD8 T cells, showed an enhanced expression of late activation marker CD25 and exhaustion marker PD‐1. Importantly, SARS‐CoV‐2 infection increased the percentage of T follicular helper– and germinal centre B–like cells in the blood. The parameters in COVID‐19 patients remained unchanged across various age groups. Therefore, we demonstrated that the T and B cells are activated naturally and are functional during SARS‐CoV‐2 infection. These data provide evidence that the adaptive immunity in most patients could be primed to induce a significant immune response against SARS‐CoV‐2 infection upon receiving standard medical care.

## 1 INTRODUCTION

A severe pneumonia‐associated respiratory syndrome began in Wuhan, China, in December 2019, which was subsequently declared as a public health emergency of international concern by WHO. The novel coronavirus strain was officially named severe acute respiratory syndrome coronavirus 2 (SARS‐CoV‐2).^1^, ^2^ Coronavirus infections, such as severe acute respiratory syndrome (SARS) and Middle East respiratory syndrome (MERS), can cause severe respiratory diseases.^3^, ^4^ SARS‐CoV‐2 is an enveloped positive‐strand RNA virus, which belongs to the same family as SARS‐CoV and MERS‐CoV based on genome similarity, *Coronaviridae*.^2^, ^5^, ^6^, ^7^

A number of studies have demonstrated that the adaptive immunity responds to coronavirus infections and is required for efficient clearance of the virus. In patients infected with SARS‐CoV, the acute phase of infection is associated with a severe reduction in T cell numbers in the blood, involving a dramatic loss of CD4 and CD8 T cells in comparison to healthy control individuals.^8^, ^9^ This suggests that SARS‐CoV infection impairs cellular immunity in the early stages of the disease. With the prolonged recovery time of SARS‐infected patients, expression of activated T cell markers, such as CD69 and CD25, decreases,^10^, ^11^ indicating that T cell activation in response to the virus is impaired.^12^ With the improvement of the disease, the ratio of CD4 to CD8 T cells increases, indicating that CD4 T cells recover faster than CD8 T cells.^13^ In addition, out of the 92% of cured SARS patients whose B cells initially declined and then increased or continued to increase during the course of the disease, only 8% had a constant or decreasing cell count.^14^ Similar to SARS patients, leucopenia and lymphopenia are also observed in MERS patients, albeit to a lesser degree than that observed in SARS patients. A clinical study showed that 14% of MERS patients had leucopenia, while 34% of the patients had lymphopenia.^15^ MERS‐CoV‐infected patients that exhibited distinctively high frequencies of MERS coronavirus–reactive CD8 T cells were associated with severe/moderate illness, whereas CD4 T cell response was minimally detected at this stage. In the convalescent phase, a moderate increase in CD4 T cells was detected.^15^

Currently, very few studies have reported that COVID‐19 patients develop lymphopenia and exhibit an increase in pro‐inflammatory cytokines in severe condition.^16^, ^17^, ^18^ The information on changes in immune cells and their functions in response to SARS‐CoV‐2 infection is still very limited. Based on the fact that T and B cells respond to infections and play critical roles in defending against virus infections, a systematic study on the changes in T and B cells in COVID‐19 patients will help uncover the immune response against SARS‐CoV‐2 infection and will also provide insights for COVID‐19 diagnosis and treatment.

A new study showed that people infected with betacoronaviruses including SARS‐CoV and MERS could establish T cell immunity to nucleocapsid protein (NP).^19^ The analysis of blood samples of 14 COVID‐19 patients displayed a strong correlation between neutralization antibody titres and the numbers of virus‐specific T cells.^20^ Wen *et al* reported that during the recovery stage of COVID‐19, plasma cells underwent a significant increase, whereas naïve B cells decreased remarkably.^21^ Several studies reported that SARS‐CoV‐2 elicits a robust B cell response, as evidenced by the detection of virus‐specific IgM, IgA and neutralizing IgG antibodies (nAbs) in the days following infection.^20^, ^22^ Importantly, Zost and colleagues identified several human monoclonal antibodies (mAbs) targeting the spike (S) glycoprotein, which exhibited potent neutralizing activity and fully blocked the receptor‐binding domain of S (SRBD) from interacting with human ACE2 (hACE2).^23^ In addition, two of the most potently ACE2 blocking mAbs have been proven to protect rhesus macaques from SARS‐CoV‐2 infection.^23^ However, further studies will be required to identify, design and synthesize antibodies and drugs targeting SARS‐CoV‐2.

In this study, we analysed the blood samples from 18 healthy donors (HDs) and 38 patients and focused on the characterization of adaptive immune cell populations and their phenotypes upon SARS‐CoV‐2 infection. We showed that upon infection, lymphocyte percentage declined, CD4 and CD8 T cells percentage within the lymphocyte population remained unchanged, and B cell percentage was relatively increased. CD4 and CD8 T cells exhibited a mild and strong activation phenotype. Notably, the percentage of T follicular helper (Tfh)– and germinal centre B–like (GCB‐like) cells increased. Similar phenotypes among the patients in various age groups indicate that aged individuals are also capable of responding to SARS‐CoV‐2 infection. Our data support the notion that adaptive immunity could be normally activated, and it could defend against SARS‐CoV‐2 infection.

## 2 MATERIALS AND METHODS

### 2.1 Ethics statement

This study was approved by the Research Ethics Commission of the Eighth Hospital of Xi'an (20190730‐1346) with a waiver of informed consent due to a public health outbreak investigation. Information regarding all the cases was taken from the Eighth Hospital of Xi'an (Xi'an, Shaanxi Province), a designated hospital for the COVID‐19 by the local authorities.

### 2.2 Patients

From 18 February to 4 March 2020, 18 healthy controls and 38 confirmed COVID‐19 patients were included in the study. Patients were diagnosed and admitted in accordance with the guidelines of the national health commission of China. All the 38 patients were diagnosed with SARS‐CoV‐2 infection using the RT‐PCR test on throat swab specimens. The study included 23 male patients (60.53%) and 15 female patients (39.47%) with median age of the patients being 39.06 ± 4.26 years (Table 1).

**TABLE 1:** Characteristic analysis of COVID‐19 patients and healthy donors

Sample information		
	Gender	Age
HD	9/18, 50%	39.06 ± 4.26
Patients	23/38, 60.53%	45.08 ± 4.06

### 2.3 Flow cytometry analysis

The Abs used in the flow cytometry analysis were as follows: FITC anti‐human CD3 (UCHT1), FITC anti‐human TCR γ/δ (B1), APC/Cyanine7 anti‐human CD4 (OKT4), PerCP/Cyanine5.5 anti‐human CD8 (SK1), APC anti‐human CD19 (HIB19), APC anti‐human CD25 (BC96), PE anti‐human CD69 (FN50), PE anti‐human CD185 (CXCR5) (J252D4), PE anti‐human CD183 (CXCR3) (G025H7), APC anti‐human CD279 (PD‐1) (EH12.2H7), PE anti‐human CD95 (Fas) (DX2), PE anti‐human CD127 (A019D5), APC/Cyanine7 anti‐human CD45RA (HI100), PE/Cy5 anti‐human CD45RO (UCHL1), PE anti‐human CD95 (Fas) (DX2) and FITC antimouse/human GL7 Antigen (GL7). They were purchased from BioLegend. Blood cells were stained with Abs in the dark at room temperature for 15 minutes and analysed on a FACSCanto II flow cytometer (BD Biosciences). FlowJo 8 was used for data analysis.

### 2.4 Statistical analysis

The continuous variable of normal distribution is represented by mean ± standard deviation, the non‐normal distribution is represented by median [IQR], and the classified variable is represented by count (percentage). Student’s *t* test was performed for two group analysis using SPSS 22.0 software. * and ** stand for *P*<0.05 and *P*<0.01, respectively.

## 3 RESULTS

### 3.1 The percentage analysis of T and B cells in COVID‐19 patients

**FIGURE 1:**
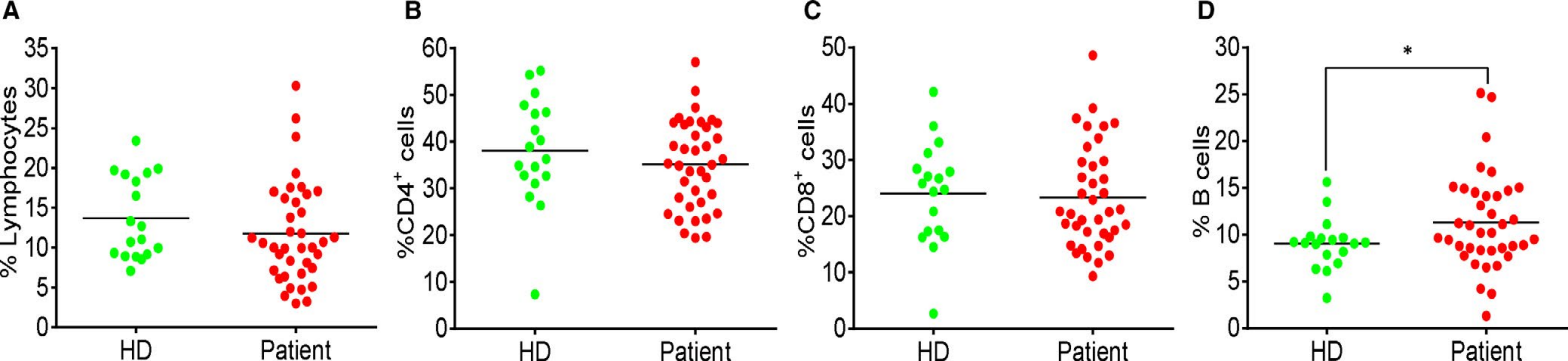
Percentage changes in T and B cells between HDs and COVID‐19 patients. (A) The percentage of lymphocytes in total blood cells. (B) The percentage of CD4^+^ T cells in lymphocyte population. (C) The percentage of CD8^+^ T cells in lymphocyte population. (D) The percentage of B cells in lymphocyte population. Each dot represents a single patient of COVID‐19 or healthy donor. *P<0.05 was considered statistically significant

To determine the change in the composition of adaptive immune cells, we compared the percentage of T and B cells in the blood samples from HDs and COVID‐19 patients using flow cytometry. Lymphocyte percentage in the whole blood was not significantly different between HDs and COVID‐19 patients, though it exhibited a decreasing trend in the patients (Figure 1A). Within the lymphocyte population, the percentages of CD4^+^ and CD8^+^ T cells were comparable (Figure 1B and C), whereas B cell percentage was significantly increased (Figure 1D) in COVID‐19 patients.

### 3.2 An activated phenotype of T cells in COVID‐19 patients

**FIGURE 2:**
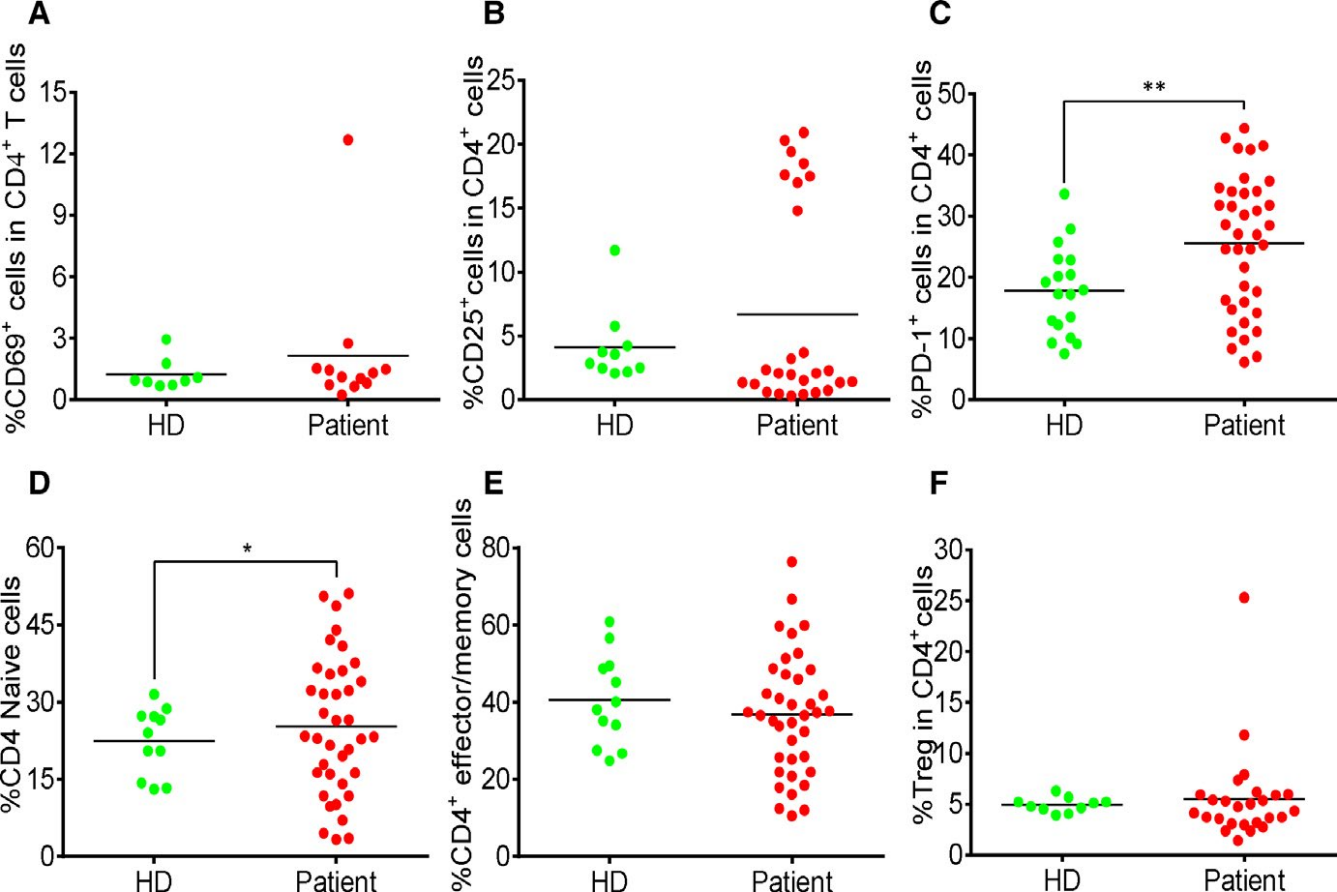
Moderate increase in activated CD4^+^ T cells in COVID‐19 patients. (A) The percentage of CD69^+^ cells in CD4^+^ T cells. (B) The percentage of CD25^+^ cells in CD4^+^ T cells. (C) The percentage of PD‐1^+^ cells in CD4^+^ T cells. (D) The percentage of CD45RA^+^CD45RO^‐^ cells in CD4^+^ T cells. (E) The percentage of CD45RA^‐^CD45RO^+^ cells in CD4^+^ T cells. (F) The percentage of regulatory T cells in CD4^+^ T cells. Each dot represents a single patient of COVID‐19 or healthy donor. *P<0.05 and **P<0.01 were considered statistically significant and extremely significant, respectively

**FIGURE 3:**
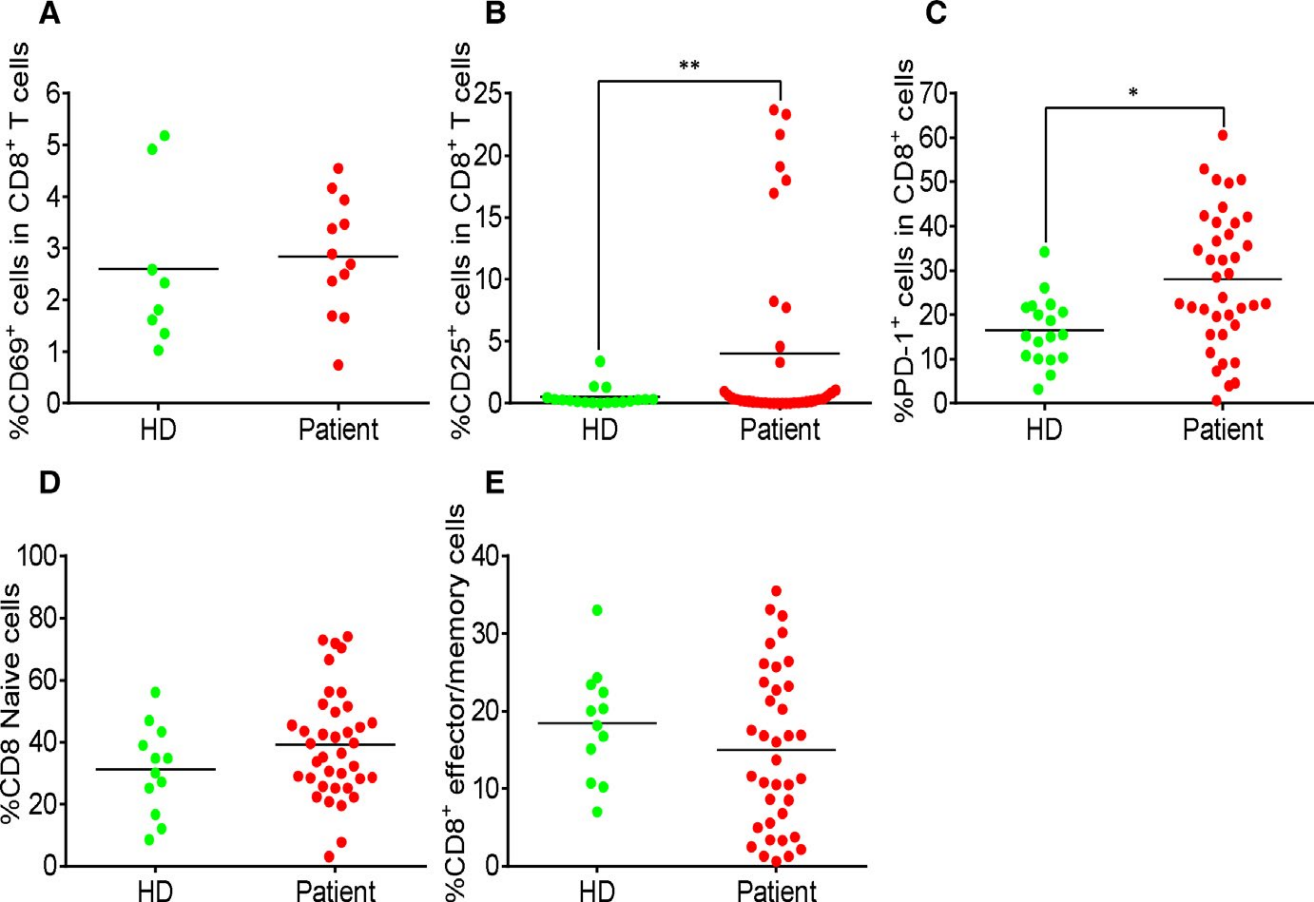
Strong increase in activated CD8^+^ T cells in COVID‐19 patients. (A) The percentage of CD69^+^ cells in CD8^+^ T cells. (B) The percentage of CD25^+^ cells in CD8^+^ T cells. (C) The percentage of PD‐1^+^ cells in CD8^+^ T cells. (D) The percentage of CD45RA^+^CD45RO^‐^ cells in CD8^+^ T cells. (E) The percentage of CD45RA^‐^CD45RO^+^ cells in CD8^+^ T cells. Each dot represents a single patient of COVID‐19 or healthy donor. *P<0.05 and **P<0.01 were considered statistically significant and extremely significant, respectively

To evaluate the T cell status in response to SARS‐CoV‐2 infection, we analysed the expression of CD69, CD25, PD‐1, CD45RA, CD45RO and CXCR3 in both CD4^+^ and CD8^+^ T cells. In CD4^+^ T cells of the COVID‐19 patients, the expression of CD69 and CD25 (Figure 2A and B) and the percentage of regulatory T cells, marked by CD3^+^CD4^+^CD25^+^CD127^‐^ (Figure 2F), were similar to that of HDs. CD25 expression was significantly up‐regulated in CD8^+^ T cell population of the patients (Figure 3B). The proportion of naive and effector/memory cells in both CD4^+^ T cells (Figure 2D and E) and CD8^+^ T cells (Figure 2E and F) of the two groups were not significantly different. PD‐1 expression was up‐regulated in both CD4^+^ (Figure 2C) and CD8^+^ T cells (Figure 3C) of the patients. The data demonstrated a weak activation of CD4^+^ T cells, but a strong activation of CD8^+^ T cells during SARS‐CoV‐2 infection.

### 3.3 An increase in germinal centre–like cells in COVID‐19 patients

**FIGURE 4:**
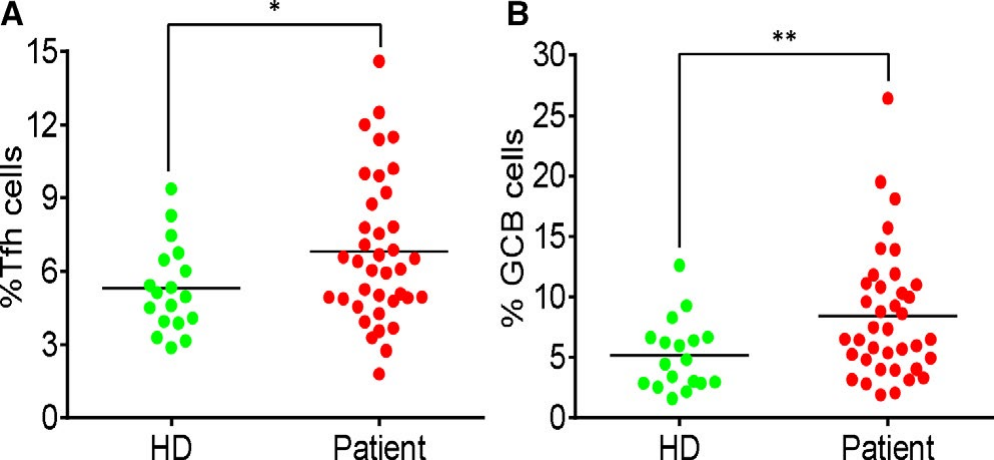
Increase of germinal centre–like cells in COVID‐19 patients. (A) The percentage of PD‐1^+^CXCR5^+^ cells in CD4^+^ T cells. (B) The percentage of Fas^+^GL7^+^ cells in B cells. Each dot represents a single patient of COVID‐19 or healthy donor. *P<0.05 and **P<0.01 were considered statistically significant and extremely significant, respectively

T follicular helper (Tfh) cells help in activation of B cells and differentiation into effector cells, production of high‐affinity antibodies and formation of germinal centres.^24^ To study whether COVID‐19 patients produced efficient adaptive immune response, we analysed the expression of PD‐1 and CXCR5 in CD4^+^ T cells and the expression of Fas and GL7 in B cells. As shown in Figure 4A and B, there was a significant increase in Tfh‐ and GCB‐like cells in the blood samples of the patients compared to that of the HD group.

### 3.4 Correlation analysis between activation signature and patient age

**FIGURE 5:**
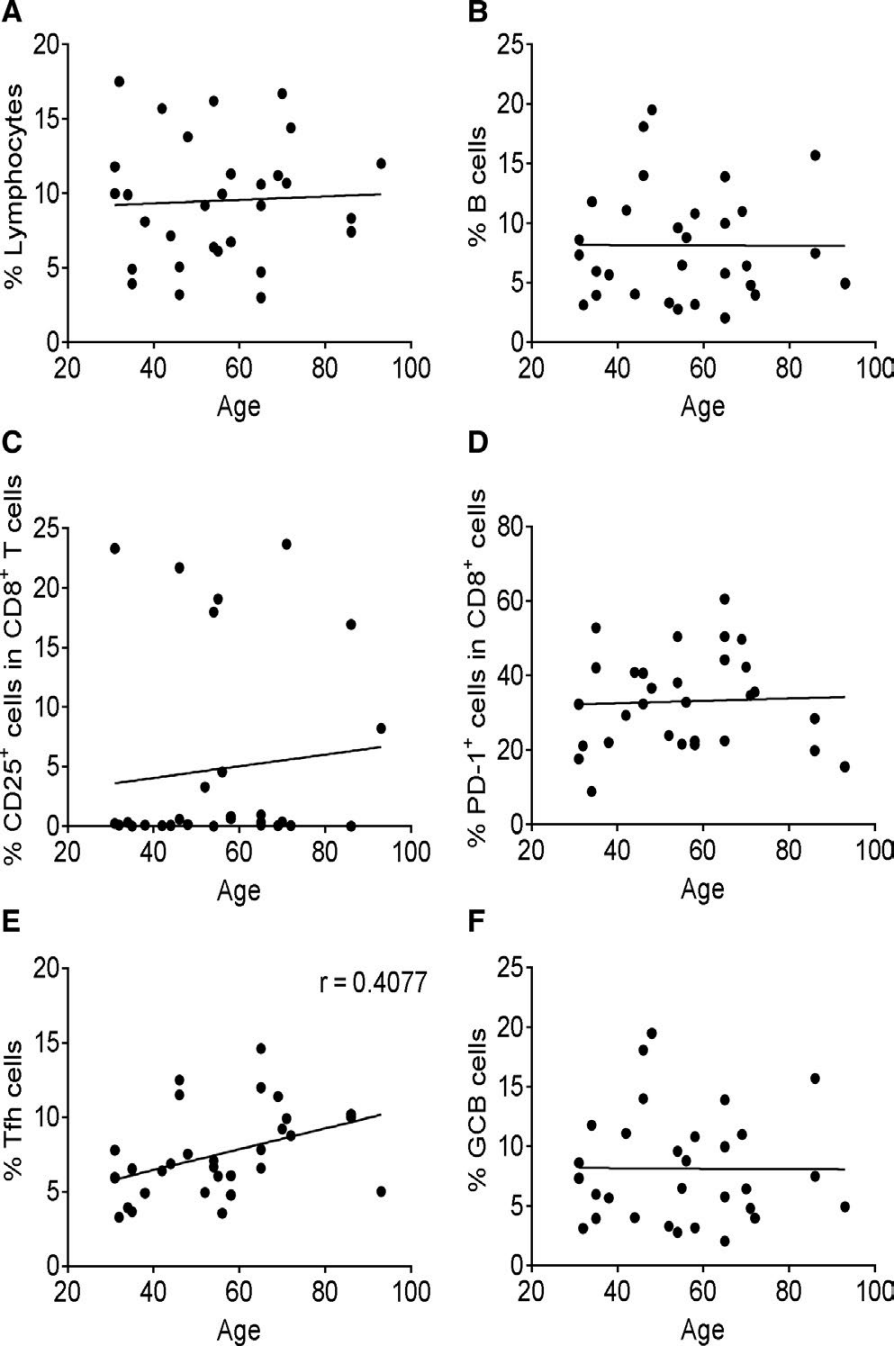
Correlation analysis between functional signature and patient age. The correlation analysis between patient age and immune parameters was performed using Pearson’s correlation coefficient. The percentage of total lymphocytes (A), B cells (B), CD8^+^CD25^+^ T cells (C), CD8^+^PD‐1^+^ T cells (D), Tfh‐like cells (E) and GCB‐like cells (F) was correlated with age in COVID‐19 patient group. Each dot represents a single patient of COVID‐19 or healthy donor. Patients under the age of 15 years were excluded

To study whether age affects adaptive immune cell populations and effector features, we performed correlation analysis between T cell activation markers and age. No dramatic change was observed with increase in age of the patients (Figure 5). The results indicate that there was no defect in CD8^+^ T cell activation and Tfh‐ and GCB‐like cell differentiation in the aged individuals infected by SARS‐CoV‐2.

## 4 DISCUSSION

SARS‐CoV‐2 infection is spreading rapidly around the world. The patients present typical symptoms of pneumonia, such as dry cough, dyspnoea, fever and bilateral lung infiltrates on imaging.^25^ Although post‐mortem study has revealed intensive inflammation in lungs of the patients, little is known about the immunological features in response to this new virus. In the present study, we have focused on characterizing adaptive immune cell populations and their phenotypes in COVID‐19 patients.

Generally, the phenotypical T and B cell subsets were found to be different with varying disease severity in COVID‐19 patients, which may be directly correlated with the viral load. Currently, the profile changes of T and B cell populations in patients suffering from COVID‐19 remain poorly established, especially in mild or moderate patients. In accordance with several previous reports, our results revealed that patients with mild or moderate symptoms were shown to have an apparent increase in follicular helper CD4 T cells (TFH) and germinal centre B (GCB) cells, while severe COVID‐19 patients displayed dysregulation of lymphocytes characterized by a profound depletion of CD4^+^ lymphocytes and subsequently B cell lymphopenia.^26^ Also, Wen and colleagues utilized single‐cell RNA sequencing to demonstrate that both CD4^+^ and CD8^+^ T cells decreased remarkably, whereas the B cells underwent a significant increase during the recovery phase of COVID‐19.^21^ Furthermore, Zhang *et al* developed an immune response phenotyping strategy based on neutrophil‐to‐lymphocyte ratio (NLR) and IgG level to stratify patients with varying disease severities and outcome, which would be helpful to guide treatment options in the clinic.^27^ Collectively, these studies provide a first glimpse into the phenotypes of T and B cell subsets associated with COVID‐19. However, the relevant conclusions need to be interpreted with caution due to the limited number of patients enrolled in these studies.^28^ Therefore, further investigation is needed to better determine the phenotype and function of T and B cell subsets in COVID‐19 patients.

Lymphopenia was observed in COVID patients in previous studies,^25^ Epidemiological investigation of coronavirus infection showed that lymphopenia is present in more than 80% of the patients, and serious decline in lymphocytes is correlated with a poor prognosis.^29^ However, we did not observe a significant decrease in lymphocyte populations in the COVID‐19 patients. This finding could be attributed to the fact that most of the patients in this study showed mild symptoms besides fever. Interestingly, following the division of patients into symptomatic and asymptomatic, we observed a decrease in lymphocytes in the symptomatic patients (data not shown). PD‐1 is a marker of exhausted T cells during chronic and acute infections.^30^, ^31^ A number of studies have shown that PD‐1^+^CD8^+^ T cells increase in the peripheral blood of patients with a variety of acute viral infections, such as HBV, HIV and Ebola virus.^32^, ^33^ In our study, the expression of PD‐1 was up‐regulated in both CD4^+^ and CD8^+^ T cells of COVID‐19 patients, which may explain the observed reduction in the lymphocyte population.

In response to viral infections, normally both CD4^+^ and CD8^+^ T cells become activated. In COVID‐19 patients, we observed mild activation of CD4^+^ T cells but stronger activation of CD8^+^ T cells based on CD25 expression. This reflects that CD8^+^ cells are major responders of COVID‐19 infection and are consistently activated. It is possible that CD4^+^ T cells may be strongly activated earlier during the infection and then revert to the quiescent state after providing helper functions, which could explain the undetected activated phenotype. This could be explained by the comparable expression of CD69, an early activation marker, in HDs and patients. CD4^+^ T cells may indeed be weakly activated in response to the virus, which warrants further studies.

During viral infections, the antigen‐specific immune response is executed by Tfh and GCB cells. Tfh cells help B cell differentiate into antigen‐specific effector cells to produce high‐affinity antibodies and facilitate GC formation,^24^ which are essential for inducing efficient virus clearance. In COVID‐19 patients, there was an increase in both Tfh‐ and GBC‐like cells in the blood, which reflected that an antigen‐specific response can be activated upon SARS‐CoV‐2 infection.

Elderly individuals typically exhibit a reduction in the lymphocyte populations and a weaker ability to defend against viral infections.^34^ In our correlation analysis, we did not observe a significant correlation between lymphocyte proportions, effector features and age. Our data suggest that the specific populations of T and B cells for SARS‐CoV‐2 are reserved in aged individuals; this needs to be proven by repertoire sequencing analysis of T cell and B cell receptors in elderly patients.

In summary, our study shows that SARS‐CoV‐2 could induce relatively normal adaptive immune response in patients. Most people across different age groups are capable of mobilizing the adaptive immune cells, and activating cellular and humoral immunity to defend against the virus with sufficient medical care and anti‐viral treatment.

## CONFLICT OF INTEREST

We declare no competing interests.

## AUTHORS’ CONTRIBUTIONS

Xiaofeng Yang, Xiaobo Zhou, Lei Lei, Xingzhe Zhang and Dan Zhang: Data analysis and writing of the manuscript. Tongxin Dai, Hongbo Qian, Rui Guo and Yaling Guo: Sample collection and information. Lin Shi and Yanbin Cheng: Data analysis discussion. Jinsong Hu: FCAS analysis. Baojun Zhang: Generation of idea, experiment design and writing of the manuscript. All authors agree to be responsible for their own part of the work. Xiaofeng Yang: Data curation (lead). Tongxin Dai: Data curation (lead). Xiaobo Zhou: Data curation (equal). Hongbo Qian: Data curation (equal). Rui Guo: Data curation (equal). Lei Lei: Data curation (equal). Xingzhe Zhang: Data curation (equal). Dan Zhang: Data curation (equal). Lin Shi: Formal analysis (equal). Yanbin Cheng: Formal analysis (equal). Jinsong Hu: Methodology (lead). Yaling Guo: Data curation (lead). Baojun Zhang: Project administration (lead); Writing‐review & editing (lead).

## Supporting information

Fig S1

## Data Availability

All data, models and code generated or used during the study are available in the submitted article.
