# Supplementary figures and images for: Naturally activated adaptive immunity in COVID‐19 patients
Source: J Cell Mol Med. 2020 Sep 25;24(21):12457–63. doi: 10.1111/jcmm.15771 (PMC7537162; doi:10.1111/jcmm.15771)

Supplementary Figure. The gating strategy depicted was used in all flow cytometric analyses.

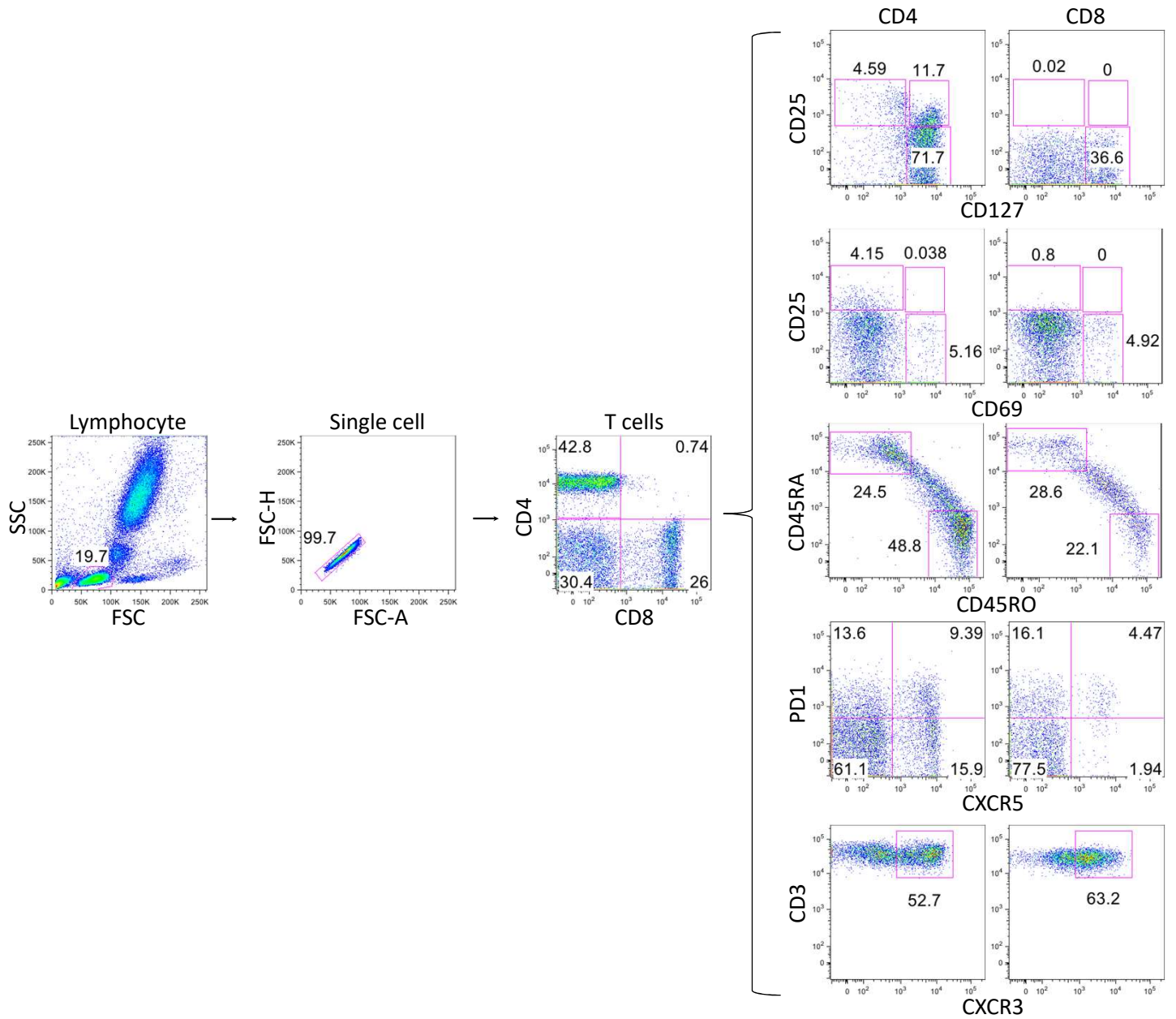

Supplement: Supplementary file 1 — Fig S1 [file JCMM-24-12457-s001.pdf]
